# Adapting SARS-CoV-2 vaccination delivery in England to population needs: a thematic analysis of providers and commissioner’s perceptions

**DOI:** 10.1186/s12913-023-09350-6

**Published:** 2023-05-01

**Authors:** Sharif Ismail, Tracey Chantler, Pauline Paterson, Louise Letley, Sadie Bell, Sandra Mounier-Jack

**Affiliations:** 1 Department of Global Health and Development, Faculty of Public Health and Policy, London School of Hygiene & Tropical Medicine, 15-17 Tavistock Place, London, WC1H 9SH UK; 2 Department of Infectious Disease Epidemiology, Faculty of Epidemiology and Population Health, London School of Hygiene & Tropical Medicine, London, WC1E 7HT UK; 3 UK Health Security Agency, 61 Colindale Avenue, London, NW9 5EQ UK

**Keywords:** Vaccine, SARS-CoV-2, Primary care, Equity, Vaccination delivery, Service delivery

## Abstract

**Background:**

A national SARS-CoV-2 vaccination programme was implemented in England from 8th December 2020, adopting a series of local level service delivery models to maximise rollout. The evidence base informing service design programme at inception was limited. We examined the real-world implementation of the programme through an assessment of sub-national providers’ and commissioners’ perspectives on the service delivery models used, to strengthen evidence on the acceptability, effectiveness and efficiency of the service delivery approaches used for SARS-CoV-2 vaccination in England or elsewhere.

**Methods:**

Qualitative, cross-sectional analysis based on semi-structured interviews conducted with 87 stakeholders working in SARS-CoV-2 vaccination delivery across four regions in England. Localities were selected according to geography and population socio-economic status. Participants were purposively sampled from health service providers, commissioners and other relevant bodies. Interviews were conducted between February and October 2021, and transcripts were thematically analysed using inductive and deductive approaches.

**Results:**

Various service delivery models were implemented over the course of the programme, beginning with hospital hubs and mass vaccination sites, before expanding to incorporate primary care-led services, mobile and other outreach services. Each had advantages and drawbacks but primary care-led models, and to some extent pharmacies, were perceived to offer a better combination of efficiency and community reach for equitable delivery. Common factors for success included availability of a motivated workforce, predictability in vaccine supply chains and strong community engagement. However, interviewees noted a lack of coordination between service providers in the vaccination programme, linked to differing financial incentives and fragmentated information systems, among other factors.

**Conclusion:**

A range of delivery models are needed to enable vaccine rollout at pace and scale, and to mitigate effects on routine care provision. However, primary care-led services offer a tried-and-trusted framework for vaccine delivery at scale and pace and should be central to planning for future pandemic responses. Mass vaccination sites can offer delivery at scale but may exacerbate inequalities in vaccination coverage and are unlikely to offer value for money. Policymakers in England should prioritise measures to improve collaboration between service providers, including better alignment of IT systems.

**Supplementary Information:**

The online version contains supplementary material available at 10.1186/s12913-023-09350-6.

## Introduction

The UK was the first country to launch a national SARS-CoV-2 vaccination programme on 8 December 2020 [1]. Vaccine deployment initially targeted health and social care workers (HSCWs) and those aged 80+, eventually moving down an initial list of nine priority groups, totalling some 27 million people, in accordance with advice from the Joint Committee on Vaccination and Immunisation (JCVI) [2]. From April 2021, the programme was extended to three more priority groups, covering those aged 18–49 without significant comorbidities, and subsequently to younger age groups [3].

The staggered deployment of the programme reflected evolution in its aims over time. Initially (December 2020 onwards), the primary goal was to reduce mortality from COVID-19 and reduce pressure on the NHS and the wider social care system [4], but later expansion to lower risk population groups emphasised reductions in disease morbidity and mortality. By the end of December 2021, reported SARS-CoV-2 vaccination coverage for those aged 12 + was 90.1% for first dose and 82.5% for second dose [5, 6].

Governance and delivery arrangements for the SARS-CoV-2 vaccination programme contrasted in important ways with those in operation for routine vaccinations under “normal” conditions. At the time during which this study was conducted, recommendations for the national, routine vaccination schedule and programme design were provided by JCVI with specialist support from Public Health England (PHE) – a system that also applied for SARS-CoV-2 vaccination. The National Health Service (NHS) in England, and NHS Improvement (NHSE&I) commissioned routine vaccination services from primary care providers (GP practices), who delivered the vast majority of routine childhood and adult vaccines [7]. Routine vaccinations could be administered by doctors, nurses and healthcare assistants, making use of a series of regulatory mechanisms: National Protocols (NP), Patient Group Directions (PGD) or Patient-Specific Directives (PSD) (see **Appendix ****2**). Regulatory aspects and safety monitoring for adverse events linked to vaccine administration were handled by the Medicines and Healthcare products Regulatory Agency (MHRA).

England’s SARS-CoV-2 vaccination programme was national, population-wide, and implemented over a compressed timeline and in the context of significant vaccine supply and storage constraints. In contrast to routine vaccination delivery, the COVID-19 Vaccine Delivery Plan published in January 2021 set out a three-pronged approach to delivery spanning mass vaccination sites, acute hospitals, and local vaccination services through primary care networks (PCNs) and, later on, pharmacies [1]. Concurrent use of multiple service models was designed to ensure vaccine delivery to as many people as quickly as possible. Approaches evolved over the first year of the programme, linked to changes in population prioritisation, availability of new vaccine products, and other factors (see timeline in **Appendix ****1**). Data indicate large variations in proportionate uptake by delivery model by the 31st October 2021: 28% of front-line health workers received vaccine doses through hospital hubs (where early supply of vaccines was available), compared to 2% of those aged 75–79; uptake through mass vaccination centres was proportionately highest among those aged 55–64 [8].

Prior to launch, there was little evidence on how to develop and implement national vaccine programmes of this scale under emergency conditions. Experiences from the Influenza A/H1N1 pandemic in 2009 highlighted challenges to delivery through mass vaccination centres, suggesting that primary care-based delivery was more likely to deliver positive results [9–12]. Little evidence has so far been published on the acceptability, effectiveness and efficiency of SARS-CoV-2 vaccination organisational delivery models [13–19]. This study examined the real-world implementation of the SARS-CoV-2 vaccination programme in England through an evaluation of service delivery models from the perspective of providers and commissioners, to inform onward programme planning and long-term pandemic preparedness in England and internationally.

## Methods

This was a qualitative study using in-depth, semi-structured interviews with stakeholders involved in the delivery of the vaccination programme in England. Data were analysed using thematic analysis.

### Site and interviewee selection

We focused on regional and local organisations involved in the delivery of the SARS-CoV-2 vaccination programme in four National Health Service (NHS) regions, representing varied geographies and socio-demographic characteristics (urban vs. rural, and indices of socioeconomic deprivation). Local areas in each region were selected by primary care Clinical Commissioning Group (CCG), or Local Authority (LA), and respondents were identified through snowball sampling with support from Public Health England (PHE) and the local NIHR Clinical Research Network. Immunisation commissioners at regional and local level, local public health professionals and vaccination providers’ staff in each selected locality were interviewed.

Interviews were performed by four core team members (TC, PP, SB and SMJ), all with extensive prior experience and research training in the application of qualitative research methods. A majority were carried out between April-June 2021. Because of the continual evolution of the vaccination programme, interviews continued (albeit in smaller numbers) until October 2021 at which point the study investigators judged that saturation around major themes had been reached.

### The conduct of the interviews

Prospective study participants were contacted by email and provided with an information sheet regarding the purpose of the work and intended use for data gathered. Staff who consented to participate were interviewed once. Interviews were performed primarily remotely (via Zoom/MS Teams), and in some cases face-to-face in their place of work. Interviews lasted on average one hour and were audio recorded with participant consent. Five SARS-CoV-2 vaccination clinics were also observed for details of site design and the process of vaccination, and field notes made accordingly. A semi-structured interview topic guide was used to inform discussions with participants, with probing around specific topics where appropriate (see **Appendix ****3**). Interviewees were asked about their experience of SARS-CoV-2 vaccination delivery, characteristics of the service models used, the nature of collaboration between actors involved, and what facilitated implementation or created challenges to it.

### Analysis

Interviews were transcribed verbatim and analysed using a thematic framework using qualitative analysis software (NVivo version 11, QSR International Pty Ltd., Melbourne, Australia). Coding was performed by the four core members of the research team. The thematic framework was developed iteratively by TC, PP, SB and SMJ using the stages outlined by Braun and Clarke (2006) [20]. The initial version of this framework was based on a combination of a rapid review of literature on delivery models applied in preceding pandemic responses, and discussions with collaborators at UKHSA. Major domains are outlined in **Appendix ****4**. Initial codes were piloted by the four researchers involved in data collection (TC, PP, SB and SMJ). We then used an inductive and deductive approach to the analysis, allowing new themes to emerge beyond this initial theoretical framework, and developing and triangulating findings through bi-weekly meetings involving the research team.

### Ethics

The study received full ethics approval from the Observational Research Ethics Committee of the London School of Hygiene & Tropical Medicine (22,655) and the NHS Health Research Authority (20/HRA/5615).

## Results

87 respondents were interviewed and 5 vaccination clinics observed between 1 February and 19 October 2021 (Table 1). None dropped out during the course of the study. Where quotes are provided in the sections that follow, the category of respondent is indicated by a code following each quotation according to the following notation: region_participant number_participant category (e.g. *R1_1_GP*). The participant categories are: CCG (clinical commissioning group); COM (commissioner); CSO (civil society organisation); GP (general practitioner); LA (local authority); MASSVAC (mass vaccination centre); AT (acute NHS trust); PHARM (pharmacy); and POP (pop-up provider).

**Table 1:** Breakdown of interviewees by category and organisational affiliation. The first and final columns in the table include summary details of the locality in which interviews were carried out

Region	Providers					Commissioners		Local authorities	Other	Total	Description of study sites
	GP	Mass Vaccination Site	Pharmacy	Pop-up provider	NHS Trust	CCG	Other				
North of England	2	0	0	0	0	3	1	**5**	**2**	**13**	Urban deprived; semi-rural
East of England	0	1	0	0	3	3	2	**3**	**0**	**12**	Mostly rural
South of England	9	3	0	0	0	5	6	**3**	**2**	**28**	Mostly rural and semi-urban
London	4	8	2	2	2	6	5	**5**	**0**	**34**	Urban deprived
**Overall total (interviews)**	**36**					**31**		**16**	**4**	**87**	
Site visits	0	2	1	2	0			0	0	5	

### Characteristics, strengths and weaknesses of vaccine delivery models

This section outlines strengths and weaknesses of the main delivery models (see in addition, Table 2).

**Table 2:** Typology of the key vaccination delivery models used in England for the SARS-COV-2 vaccine rollout, up to the end of October 2021

Models	Description	Positive features	Challenges
Hospital hubs	• Block contracted by NHSE to serve priority groups, based on estimated utilisation	• Ability to maintain super cold storage for mRNA vaccines early in the programme • Effective reach into registered patient populations through modified call-recall	• Logistical difficulties in ensuring access for some priority groups, especially older, vulnerable patients
Mass vaccination sites	• Block contracted by NHSE based on estimated utilisation • Located based on availability of appropriate sites using sites including conference centres, stadiums and others • Staffing from local service providers and trained surge capacity • Appointment booking through NBS	• Scale of vaccine administration possible • Local modifications to the basic model (using outreach) improved uptake among vulnerable groups	• Access issues for some cohorts due to e.g. cost of transport, reluctance to use public transport for those shielding • Poor appointment uptake as booking required digital access • Perceived risk of contracting COVID-19 on-site • Lower trust from patients than primary care models
Primary care delivery models	• Local models grouping together a number of GP practices of varying sizes • Location based on existing premises within a PCN’s area • Contracted on fee-for-service basis • Multiple variations including hub-and-spoke approaches, ring-fencing specific clinic days within each practice, or nominating a single practice within a PCN to focus entirely on vaccination	• High uptake especially among vulnerable and ethnic minority groups - capitalising on trust and established relationships • Less prescriptive approach to service design permitted flexibility to develop services better tailored to local needs, but also to local capacity	• Sidelining of regular clinical care contributed to withdrawal of some PCNs from later phases of the program • Slow path to approval for delivery of Pfizer vaccines in the community • Large variations in capacity across the country contributed to variations in service delivery approach
Pharmacies	• Selected in discussion with PCNs based on identified geographical gaps in provision • Contracted through regional pharmacy commissioning teams on fee-for-service basis • Appointment booking through NBS	• High uptake given geographical footprint, long opening hours, community links and presence of staff with relevant language skills	• Participation in the programme limited by target minimum vaccination rate of 1000 doses per week, shortages of staff, space and cold chain capacity • Initial inability to pre-book or re-book patients through NBS made appointment management challenging
Roving and outreach models	• Tailored delivery, mostly developed as partnerships between PCNs and local public health teams, but delivered by PCN staff • Varied outreach models including fixed, temporary sites (e.g. supermarkets) and mobile approaches	• Ability to target specific communities or vulnerable groups for which access was limited or uptake low • Community engagement seen as effective	• Cost-effectiveness unclear – best used as highly targeted service delivery approaches

Abbreviations: NBS, National Booking System; NHSE, NHS England; PCN, Primary Care Network

#### Hospital hubs

Hospital hubs were the first to vaccinate priority groups such as HSCWs and those aged 80+, and were introduced because the Pfizer vaccine (BNT162b2) required a special ultra-cold chain for effective storage that was only available in these settings initially. Participants noted that high levels of circulating infection at the time and vulnerability of the first elderly recipients generated both logistical challenges and anxiety. Hospital hubs were wound down when mass sites and PCNs became operational as SARS-CoV-2 vaccination delivery sites from January 2021, and practical insights on these sites from participants were limited.

#### Mass vaccination sites

Mass vaccination sites were set up in most local CCGs using large, existing venues (see Table 2), or occasionally from scratch using temporary planning applications made under COVID-19 emergency legislation. Although nominally open to everyone, in reality, accessibility for those with disabilities and older age, ethnic minority and socio-economically deprived groups was variable: “*It was pretty clear to us from the first weeks that the Asian, black and minority ethnic groups were not being represented in the people who walked into the vaccine centre, they were… mostly white British” [R4_48_MASSVAC].* These factors contributed to the perceived underutilisation of mass sites, and in some areas, perceived inefficiency resulted in the early closure of mass vaccination sites.

Interviewees reported that for mass vaccination centres in large urban areas, a large proportion of vaccines were ultimately delivered to those living out of area (defined as people living within 100 miles of the site, later reduced to 45 min from the home address). They also noted a clear patient preference for hyperlocal services rather than mass vaccination:

“…*In London, certainly within [Urban LA] partly driven partly by poverty and probably driven by the very local nature of what people can access, [residents] won’t travel more than 500 yards from where they live… We… knew that a localised service was going to be what they needed… particularly for those which are a bit more vaccine hesitant, and it was a real barrier in our view… not having something which was very local*.” *[R4_44_GP]*.

Some sites tried to use spare capacity and by tailoring services. Measures included hiring taxis to bring patients in for their appointments; inviting unvaccinated patients from newly announced priority cohorts using their patient lists; organising roving clinics targeting specific vulnerable groups such as those in renal units or those who were homeless; and redeploying staff to local pop-up clinics (either ad hoc or at fixed times each week), many located in faith and community settings.

#### Primary care delivery model: GP-led vaccination delivery

GP-led vaccination delivery incorporated many different models (Table 2). These sites could administer up to 3,000 vaccinations a day and might also provide roving (outreach) services. With additional funding, some PCNs also took charge of vaccination in care homes, and some operated pop-up clinics.

Decision-making concerning the set-up of GP-led delivery was described as collegial, pragmatic and based on assessment of available capacity and practices’ willingness to lead. Collaboration was more likely when a group of GP practices had a history of working together, and when it made economic sense to rent a large site collectively. Other factors promoting GP collaboration included the local availability of large clinic spaces (for higher patient flow), and a shared data management system for inviting patients.

General Practice was perceived as well placed to deliver SARS-CoV-2 vaccines: “*GP has got a track record of delivering mass vaccination programmes [flu] through vaccination programmes, we’ve got well trained staff, we’ve got an infrastructure and we have a lot of trust, which is so important, particularly for a new vaccine” [R4_32_GP].* Other interviewees contrasted the “*conversation with a GP* [who] *knows about your health with a mass site which will provide you with a leaflet” [R4_39_CCG]*, particularly for older patients who have a “*close link with their GP*” *[R3_61_MASSVAC]*. The call and recall work done by GP staff – when combined with outreach – was considered uniquely successful in ensuring high uptake: *“That is just not something that happens to a place that’s served only by pharmacies or a mass vaccination site” [R1_13_CCG]*.

However, regulatory barriers to GP-led delivery were significant and slowed the integration of providers into the programme: “*[GP practices] have to fill in a form every time they want to pick up a vial… which for small practices is… an ask, and also that creates an administrative burden centrally as well*” *[R4_44_GP]*. Use of BNT162b2 in GP settings was initially limited by government officials’ concern at the perceived risk of cold-chain failure, and though the introduction of the AstraZeneca (ChAdOx1) vaccine transformed this situation, it took time for dispersal regulations to be updated accordingly. Many primary care providers also lamented that single-practitioner GPs had not been authorised to vaccinate their own patients despite established patient relationships that would have helped address hesitancy including among ethnic minority groups.

There was a recognition that individual practice capacity varied considerably, influencing the kind of offer that GP-led services could provide, for example in terms of ability to vaccinate housebound patients. Resource trade-offs were also common: as vaccination-related workload increased, some practices found they could only offer emergency appointments. In other PCNs, practices were only involved part-time and maintained business as usual on top of SARS-CoV-2 vaccination delivery.

As the vaccination programme extended delivery to younger cohorts, some PCNs scaled back SARS-CoV-2 vaccine to focus on regular care. This led to some CCGs “*strong-arming”* GP practices to continue participating in the programme despite a rising clinical caseload: “*If general practice just steps back because they just haven’t got the time or energy to do so, they’re fed up with being criticised for not having appointments for routine stuff” [R4_32_GP]*. Where practices did pull out, this was usually due to a desire to go back to “*being GPs*”.

#### Pharmacies

Although part of the local vaccination component of the government’s delivery plan, pharmacies were perceived as an afterthought in the provider landscape: by July 2021 less than 10% of flu-vaccinating pharmacies in one region were involved in the programme. Reasons for their late introduction included capacity constraints (in terms of staffing and cold chain capacity), but the *“complex”* authorisation and assurance process required, involving a site visit by the NHS regional pharmacy commissioning team. They also had to use the National Booking System (NBS) and – like mass vaccination sites – were not able to pre- or re-book patients, which limited the effectiveness of outreach to the local community, although some LAs and pharmacies later developed work-arounds for this.

Once pharmacies were onboard, their accessibility was seen as a strong asset: “*in the last year particularly community pharmacy has been the one facet of primary care that has remained open throughout” [R4_31_COM]*. Some pharmacies were able to maximise uptake by serving particular vulnerable groups (e.g. methadone users) and responding to younger cohorts’ preference for convenience. They also leveraged existing community networks: one pharmacist proactively reached out to young unvaccinated men in local pubs, for example. Many respondents saw local pharmacies as pivotal to access for multi-ethnic urban populations.

#### Roving and outreach models

Roving models were used by multiple providers to bring vaccination closer to communities facing barriers such as access to health services, digital- or language-based exclusion, doing so using trusted spaces and with relevant language support, and were developed in collaboration with local public health teams. Delivery was usually via PCNs, but sometimes mass vaccination sites. Examples included: (i) taking vaccines into the community (e.g. using buses); (ii) setting up temporary sites in busy communal spaces; (iii) going to locations used by the targeted communities (community centres, places of worship, Roma travellers’ sites, workplaces, and music festivals). Vaccination could also be combined with other services to address vaccination-induced anxiety such as relaxation techniques or cognitive behavioural therapy, and was in some cases incentivised (e.g. giving free drinks). Two sites operated an intergenerational pilot model where the whole family could come and access vaccination, irrespective of where they fell on the priority group list.

As regulations and vaccine supply became more amenable to off-site administration, all local areas started developing “*hyper-local*” clinics. These were aimed at facilitating access for specific communities and those who did not have access to booking systems (e.g. no NHS number, no access to booking website) or were not registered with GPs. Community involvement was seen as key for success. While these models were seen as important in reaching under-immunised communities, respondents noted that “*they’re also very resource intensive and time consuming, so it’s not actually possible to do pop-up sessions for everyone, everywhere” [R3_70_CCG]*.

### Common success factors for delivery across models

#### People

Many interviewees highlighted the importance of staff motivation (described as “immense goodwill”), a strong team ethic and flexibility in adapting to challenges in service delivery. One manager described being overwhelmed with volunteers at the start of the programme; others noted the additional capacity opened up by deployment of military staff to mass vaccination sites. As understanding of regulations around delivery improved, task-shifting also created new opportunities for capacity expansion, especially for PCNs: *“we’ve gradually shifted from being mostly doctor and nurse led with the pharmacist drawing up the vaccines, to increasingly using non clinicians to actually do the jabbing” [R4_32_GP]*. Individual innovation, and a willingness to capitalise on this, also played a role in, for example, strengthening outreach: *“tak[ing] out vaccine to a soup kitchen and things… [with] some of the other vaccines we might be able to do that but it was very much one of our local GPs said ‘I want to try and do this’, and it’s like, yes, let’s give it a go” [R3_64_COM].* However, the extraordinary pressures under which staff had been working were recognised as unsustainable over the long-term: “*there is potentially a major challenge here in terms of maintaining the morale of the workforce and the energy in the workforce. People are really tired” [R4_32_GP]*.

#### Supply chain predictability

Supply management was described as a huge logistical challenge and getting the balance right took time. Push mechanisms for vaccine deployment from the centre dominated early in the programme, sometimes leading to oversupply for smaller providers who struggled to administer doses quickly enough: “*I think it was like a Friday and they expected… us to take delivery on the Monday and then run the clinics on the Tuesday/Wednesday… so, that’s bringing all the teams over the weekend to book 1,000–1,200 patients, to set up what the clinic is going to look like, get the staff ready” [R3_69_GP].* Local allocation improved over time as providers were given a greater say in how and where doses were distributed to, but overall the approach was described as *“feast or famine”*. In periods of shortage, mutual aid mechanisms helped overcome unpredictability in supply in some regions: *“Look, I’ve got some Pfizer that’s going out of date on Saturday but I’m not going to be able to use it in time, do you know anyone who might use it?” [R2_24_CCG].*

#### Community engagement

Effective community engagement was consistently identified as central to success irrespective of the delivery model. Community ownership was important for improving uptake in populations for whom barriers to access were known to be significant: *“So we’ve done vaccinations in the mosques already and what we’ve found there was when the NHS was seen to be organising it… there was some uptake but it wasn’t brisk. But when we’ve handed it over to the faith leader and the community, then the uptake surged” [R3_61_MASSVAC]*. Community leaders were sometimes nominated to make mass-bookings on behalf of community members. However, comprehensiveness of engagement was variable across areas, and funding for outreach was described as “confused”, with multiple funding streams that local authorities and NHS providers could apply to. Finally, set payments per vaccine dose delivered by PCNs and pharmacies were often seen as insufficient to cover the costs of outreach delivery to vulnerable groups, once overheads were included.

### Challenges influencing SARS-CoV-2 vaccine delivery on the ground

#### Efficiency vs. maximising vaccination uptake

Although the use of multiple delivery models concurrently was deliberate, to maximise vaccine administration rates, participants acknowledged the cost to efficiency overall. Mass vaccination sites were widely perceived as poor value for money, while financial barriers to entry for other providers could be significant. One interviewee noted high start-up costs for a new GP-led service that had to use its own financial reserves to pay for delivery initially: *“it puts a huge financial pressure on… practices run differently… [we had] £360,000 in the pot that we could start off. So we paid the first few clinics out of that because it took some time before we got any payments through at all” [R3_76_GP]*.

#### System fragmentation vs. integration

Many interviewees highlighted the detrimental effect of silos in service delivery on vaccination uptake and efficiency, though integration appeared to improve over time. “*The way the delivery has worked it is very siloed, they do not look at the borough, we are doing delivery to mass vax, to pharmacy, and to primary care, they do not look at the [locality] as a whole” [R4_47_GP].* Supporting IT systems were often different and poorly inter-operational, including different appointment booking systems, staffing systems, data recording systems, and call and recall systems between PCNs and other providers. Poor visibility across booking systems limited the ability of LA teams to address emerging inequalities of access and meant that patients could be called or recalled multiple times by different providers: “*So, then we were asking our girls to phone or send text messages, and that was quite demoralising because probably about 50–60% of them had appointments at the XXX pharmacy that we had no idea about. So, it’s that kind of duplication of work which seemed a bit ridiculous” [R3_75_GP]*.

System fragmentation also meant that resources could often not be shared or pooled locally – particularly staff:

“*It’s quite difficult for us to understand capacity and supply across the system in an automated way so often, it means that someone has to send an email or we have to have a meeting where we go through [it]… by having it split into multiple systems that don’t talk to each other and don’t connect and then have a data lag on things as well, that causes quite a lot of issues for us” [R4_34_LA].*

#### Collaboration vs. competition between providers

The organisation of the vaccination programme along “delivery pillars” contributed to siloed service provision and, to some extent, the exclusion of key stakeholders including LA public health teams. Local commissioners and inequalities steering groups did their best to provide a coherent vaccination offer locally, but this did not always translate into effective collaboration between providers, because of historical legacy effects, differing financial incentives and commissioning pathways, and perceived power inequalities between professional groups.

Non-GP providers were often critical of the prominent role taken by GPs in the programme, noting that their incumbent position in routine vaccination delivery and in key governance positions created “unfair” barriers to entry for pharmacists. *“Remember, GPs, are still in charge of CCGs… So even if they can’t deliver, because they can’t deliver all those things, they can’t do pop ups…the surgeries aren’t in the right sites. All of those things, they have no incentive to make those other things happen” [R4_30_COM].*

Interviewees acknowledged that providers were competing for patients. One mass vaccination provider summarised: *“… We’ve all been fishing in the same pond. So there’s GPs fishing, there’s local vaccination centre fishing, and there is mass vaccination centres fishing,”* noting that the resulting lack of predictability in patient attendances had consequences for ability to plan staff and vaccine supplies *[R4_50_MASSVAC].* Inequalities in access to booking information contributed to this problem. GPs were criticised for use of their practice lists to “*fish in their pond*” and book in second dose appointments for patients who had received first doses elsewhere, and for “*dipping”* into succeeding cohorts on the JCVI priority list before these had been nationally authorised *[R3_85_MASSVAC]*. GPs in turn complained that the NBS put other providers ahead of them in the queue to invite new cohorts.

Differing financial incentives also exacerbated competition for patients. GPs and pharmacies were remunerated at a rate of £12.83 per vaccination while mass centres were paid through block contracts. Equally, some GPs observed that payments did not reflect the level of effort invested especially for outreach to vulnerable patients by comparison with the more routinised approach used by mass vaccination sites. *“So there is a bit of unfairness in the GP community about. We’re getting paid less to do the harder patients” [R4_39_CCG]*. GP practices saw mass vaccination sites as inefficient competitors that were “*getting in the way” [R1_1_GP]*.

#### Flexibility vs. direction in clinical protocols and guidance

Three legal mechanisms governed workforce allocations for vaccine delivery: the National Protocol (NP), Patient Group Directives (PGD) and Patient Specific Directives (PSD) (**Appendix ****3**) [21]. Interviewees highlighted discrepancies between published protocols and the position on the ground. Sometimes this was a matter of slow translation of changes in JCVI guidance into relevant protocols and supporting standard operating procedures (SOPs). The regimented approach to progression through priority groups also did not always align with supply of vaccines or staff availability locally: *“in this neck of the woods, we’d literally phoned, messaged, all our over 60s, and we’ve got vaccines sat in the fridge but we cannot go down to the younger cohorts. But we’ve got a clinic set up in two days fully staffed, with vaccine, and you’re telling us we can’t invite that cohort of patients in” [R3_77_GP]*.

Elsewhere, guidance seemed poorly adapted to operational realities. The absence of healthcare assistants from the list of workforce cadres eligible to vaccinate under supervision under PGDs, and the NP restriction of clinical supervisor roles to very senior nurses only, were both perceived as limiting delivery capacity. On the other hand, some forward-looking PCNs saw opportunities to create efficiencies because of the scale at which delivery was occurring: one pop-up clinic provider described process-mapping vaccine delivery and allocating specific tasks in assessment, vaccine preparation and administration and supporting record-keeping to individual staff members to speed up patient flow. It was acknowledged that some providers were also slow to adopt the NP when their preferred PGD model – usually GP-led – was probably less efficient and more time-consuming for patients.

## Discussion

### Summary of key findings

In our analysis of provider and commissioner perspectives on SARS-CoV-2 vaccine delivery through the national programme in England, we identified five service models with additional variations reflecting local population needs and specific features of the service landscape in different localities. Each model had strengths and weaknesses, but the superiority of primary care-led models for equitable vaccination delivery at scale and speed was a recurrent theme. Pharmacies offered an important, secondary delivery pathway but were involved relatively late in the programme. Delivery statistics support these observations: while initial planning assumptions were for 41% of vaccinations to be delivered at mass sites and 56% by GPs and pharmacies, by 31st October 2021 the equivalent figures were 21% and 71% (56% by GPs alone) for first and second dose vaccinations [8]. Overall cost per dose was substantially lower in GPs and pharmacies (£24/dose) than mass sites (£34/dose) [8].

Siloed service delivery was a consistent challenge despite (or perhaps because of) the proliferation of pathways. This occurred because of a combination of power imbalances between key stakeholders, incentive misalignment, and information flow problems – especially for appointment booking, a problem also observed in other countries [19].

Factors contributing to better vaccine uptake included strong community engagement, which was highlighted as one reason for the comparative success of PCNs, pharmacies and outreach models compared to mass sites, as shown elsewhere [17]. Nationally set protocols governing vaccination delivery created barriers to use of potentially valuable workforce cadres locally, but also allowed forward looking providers space to experiment with high-throughput delivery approaches given the unique pressures of the programme. Contracting and payment arrangements were complex, and there was a mismatch between payment levels and the actual cost of implementing some specialised forms of service delivery (particularly outreach services), on a background of comparatively low remuneration for SARS-CoV-2 vaccination in primary care in England [22].

### Strengths and limitations

This is the first study to present a national view of the real-world implementation of the vaccine delivery programme in England. Our analysis benefits from a purposive sampling approach to bring out variations in delivery approach according to geography and population. Although response bias cannot be excluded, the sampling and analysis approaches allowed for triangulation of findings across areas to strengthen confidence in the study results.

An important constraint, however, was timing: most interviews were conducted between April-July 2021 meaning that major developments before and after this time may not have been captured in detail. In addition, the focus on providers’ and commissioners’ perspectives may have resulted in potentially important insights from service users being overlooked. This is especially important in consideration of patient preferences for different service user models. The results of our study are indicative, but cannot conclusively show that the discrepancy between planning estimates and observed uptake of vaccination through the different models cited above reflect provider, commissioner and service user preferences for primary care-led models over mass vaccination sites, for example. Finally, our study did not directly address questions of cost and cost-effectiveness. Additional work will be needed to consider the cost implications of delivery through the various models described above, to better inform decision-making for future pandemic preparedness and response both in England and internationally.

### Policy and operational implications

Findings reinforce those from other studies in the UK and Germany suggesting that primary care-led delivery models (including pharmacies) should be central to future pandemic preparedness and response [9, 15]. However – as interviewees highlighted – diversion of resources to support national objectives on an emergency footing can undermine routine care. We found evidence of various strategies used by PCNs to help meet vaccination programme demands, including suspension of regular care. This is unlikely to be sustainable long-term given well-recognised pressures on primary care services [23] and the disruptive impact of the pandemic on delivery of routine care overall [24–27]. It is also unlikely that any single model will have the capacity to meet national demand alone in a future pandemic, while also ensuring equality in vaccination delivery, and for this reason a mixture of service delivery approaches is likely to be needed. Finally, any assessment of the potential transferability of findings from this study to other countries will need to consider the effect that differences in service delivery arrangements may have on outcomes, and the extent to which the trusted role of GPs in supporting routine vaccination in England historically may have had in influencing findings in this study [28].

Three further policy implications emerge from this work. Firstly, place-based approaches to commissioning local services may encourage whole-population approaches and help overcome some of the fragmentation problems seen early in the programme [29]. Secondly, from a workforce perspective, registration requirements for vaccine administration under NPs and PGDs could be amended to increase surge capacity and reduce the likelihood of burnout, without compromising on clinical supervision needs. Thirdly, a key long-term focus should be on improving integration of data systems supporting delivery. This is essential from an operational perspective to help target resource-intensive outreach models to address low uptake among vulnerable groups.

## Conclusion

National, regional and local systems in England responded innovatively to support SARS-CoV-2 vaccination rollout via multiple models, but initial reliance on mass vaccination sites provided neither the pace nor equity in delivery required. Future pandemic preparedness planning should centre primary care-led delivery approaches within a broad mix of service delivery models, acknowledging trade-offs with delivery of routine care, and address barriers to collaboration between services.

## Electronic supplementary material

Below is the link to the electronic supplementary material.

Supplementary Material 1

Supplementary Material 2

## Data Availability

Data supporting the findings of this study are available on request from the corresponding author SM-J. The data are not publicly available due to them containing information that could compromise research participant privacy/consent. *Competing interests*. Tracey Chantler, Sadie Bell, Pauline Paterson and Sandra Mounier-Jack report that they were in receipt of funding from the National Institute of Health Research while conducting this research. Sharif Ismail holds a Wellcome Trust Clinical Research Training Fellowship (215,654/Z/19/Z). Pauline Paterson also receives research funding from Place-based Climate Action Network (PCAN) and the Innovation and Technology Commission, Hong Kong. Louise Letley worked for Public Health England and UKHSA for the duration of this research.

## List of abbreviations

CCG: Clinical Commissioning Group
GP: General Practice
HSCW: Health and Social Care Worker
JCVI: Joint Committee on Vaccination and Immunisation
LA: Local Authority
NBS: National Booking System (for vaccination appointments)
NHS: National Health Service
NHSE: NHS England
NIHR: National Institute for Health and Care Research
NP: National Protocol
PCN: Primary Care Network
PGD: Patient Group Directive
PHE: Public Health England
PSD: Patient Specific Directive
UKHSA: UK Health Security Agency
