# Supplementary material for: Adapting SARS-CoV-2 vaccination delivery in England to population needs: a thematic analysis of providers and commissioner’s perceptions
Source: BMC Health Serv Res. 2023 May 1;23:417. doi: 10.1186/s12913-023-09350-6 (PMC10150662; doi:10.1186/s12913-023-09350-6)
Supplement: Supplementary file 1 — Supplementary Material 1 [file 12913_2023_9350_MOESM1_ESM.pdf]

Covid-19 vaccine delivery: Understanding providers' views on the acceptability and feasibility of vaccine delivery models

Topic Guide

v.1_25 Aug 2020, v.2_26 Jan 2021, v.3_15 Feb 2021,

1. Role in relation to the delivery of Covid-19 vaccines
2. Involvement in the development of the Covid-19 vaccine delivery strategy

   Probes/notes: Tease out how the national strategy was communicated/developed/receipt of guidance and related timing. Level of flexibility to adapt the strategy regionally/locally level, who was involved in this.

3. Delivery of the COVID-19 vaccine programme in their area

   - What vaccination sites (delivery models) are being used to deliver COVID-19 vaccines to the current priority groups *(refer to JCVI guidance)*
   - What influenced the choice of sites? How were organisations selected and commissioned to run vaccination sites? *(Probe: tenders, other recruitment)*
   - Explore how following factors are being managed - **lessons learned, good practice, key challenges**, what they will aim to improve over time.
     - <u>Places, information & bookings</u>
       - Choice of site locations/structures *(probes: rationale, availability)*
       - Accessibility for target groups – convenient and equitable *(Probes: consideration for frontline staff & working hours and clients who are housebound or in care homes, those with caring responsibilities at home, people who cannot afford transport, vulnerable groups e.g. those experiencing homelessness, those with physical and learning disabilities, prison inmates)*
       - Acceptability from target groups perspective – client centredness *(Probes: involvement of local authorities, community engagement, any issues related to vaccine hesitancy, social deprivation, cultural or other barriers)*
       - Specific interventions to outreach to vulnerable and under-vaccinated populations (*Probes: have you implemented any specific interventions to increase equity of uptake, outreach to vulnerable, specific ethnic minority and deprived populations, involvement of community groups, leaders, volunteers)*
       - Vaccine information, booking system *(Probes: what information and how provided, how are people contacted, the booking process, how is this monitored across sites)*
       - Records *(Probes: how is vaccination data recorded, what data management tools are used, who can access this data, use of any apps or similar certification systems, documentation provided to those vaccinated)*

- Prioritising
    - How is the process of prioritisation managed in your area? *(Probes: population groups, dosage/schedule/type of vaccine, questions from public, professionals)*
    - Are you developing your vaccination strategy using the **QCovid combination of risk factors** guidance? Can you explain how this translates on the ground?
    - Should vaccination sites/delivery models be adapted, as target group eligibility expands? How?

- Resources
    - Do you feel you have sufficient resources (including staff) to achieve your vaccination objectives?

4. Responsibility for the coordination of the COVID-19 Vaccine Programme (For CCGs and coordinators locally)

    - Strategic – who, which organisations is/are responsible for oversight/governance of the programme in your area?
    - Coordination and supervision mechanisms *(Probes: steering groups/committees, internal and external site monitoring)*
    - *Health inequalities task force/workstream*
    - Data capture for regional and national reporting purposes

5. How are you managing to maintain routine immunisation service during the pandemic? *(Probe: potential impacts of COVID-19 vaccine roll-out on childhood, school-age, influenza immunisation programmes, sustainability of COVID-19 human resource allocation etc.)*
6. Suggestions for improvement of the COVID-19 vaccine programme (e.g. coordination/strategy, addressing health inequalities), or anything else you would like to add?
7. Who else should we interview in your area?

Many thanks for your time
