# Supplementary material for: Adapting SARS-CoV-2 vaccination delivery in England to population needs: a thematic analysis of providers and commissioner’s perceptions
Source: BMC Health Serv Res. 2023 May 1;23:417. doi: 10.1186/s12913-023-09350-6 (PMC10150662; doi:10.1186/s12913-023-09350-6)
Supplement: Supplementary file 2 — Supplementary Material 2 [file 12913_2023_9350_MOESM2_ESM.docx]

## Supplementary Information

### Appendix 1: Timeline for the vaccination programme in England

| ***Date*** | ***Event*** |
| --- | --- |
| 2 December 2020 | JCVI defines nine priority groups for vaccination population-wide, with a focus for the first phase of the programme on older people (defined as those over the aged of 50), those defined as clinically vulnerable, and frontline health and social care staff |
| 8 December 2020 | First Pfizer vaccine (BNT162b2) administered at a NHS hospital hub in Coventry, marking the launch of phase 1 of the programme |
| January 2021 | COVID-19 Vaccine Delivery Plan published |
| 7 April 2021 | JCVI advises offering an alternative to the ChAdOx1 vaccination to all those aged under 30 without underlying health conditions, based on data on emerging risk of venous thromboembolic events |
| 26 April 2021 | Initiation of phase 2 of the delivery programme, extending vaccination with two doses to priority groups 10-12 (ages 18-49) |
| 7 May 2021 | Restriction of ChAdOx1 administration extended to those aged 30-39 |
| 14 May 2021 | JCVI advises reduction in the inter-dose interval from 12 to 8 weeks in light of spread of the Delta variant |
| August 2021 | Initiation of phase 3, extending vaccination with a single dose to children aged 16-17 |
| September 2021 | Extension of phase 3 to include children aged 12-15, for vaccination with a single dose |

### Appendix 2: Legal mechanisms underpinning SARS-CoV-2 vaccine delivery in England

| ***Mechanism*** | ***Description*** |
| --- | --- |
| National Protocol (NP) | - For single, registered health professional who carries out all steps in vaccination; or combination of a single, registered professional (providing oversight) and registered or un-registered individual(s) administering vaccine - Clinical assessment carried out be a prescriber or specified registered health professional - Actual vaccine administration may be carried out by a registered or unregistered individual appropriately trained, and with clinical supervision |
| Patient Group Direction (PGD) | - For use by a single registered healthcare professional undertaking the entire process of vaccination - The list of professional groups able to use the PGD includes dieticians, midwives, nurses, pharmacists and others listed in PGD legislation in operation in England |
| Patient Specific Direction (PSD) | - For use by individuals not covered by either the NP or PGD - Written instruction to vaccinate provided by a prescriber following a clinical assessment - Vaccination administered by a registered or unregistered individual under the clinical supervision of the person who provided the PSD (the prescriber) |

### Appendix 3: Interview topic guide

Please refer to separate PDF document.

### Appendix 4: Thematic framework domains

The starting framework for the study addressed the following thematic areas:

- Characteristics of the different delivery models;
- Drivers of change in service delivery;
- Effectiveness of models in vaccinating specific underserved groups;
- Governance and collaboration;
- Workforce aspects;
- Data and supporting systems (e.g. appointment booking and supply management);
- Efficiency and sustainability aspects; and
- Principal challenges and facilitators to delivery
